# Validity of reported retention in antiretroviral therapy after roll-out to peripheral facilities in Mozambique: Results of a retrospective national cohort analysis

**DOI:** 10.1371/journal.pone.0198916

**Published:** 2018-06-21

**Authors:** Yves Lafort, Aleny Couto, Ute Sunderbrink, Roxanne Hoek, Estifanos Shargie, Jinkou Zhao, Kirsi Viisainen, Bertha Simwaka

**Affiliations:** 1 GFA Consulting, Hamburg, Germany; 2 International Centre for Reproductive Health, Ghent University, Gent, Belgium; 3 Ministry of Health, Maputo, Mozambique; 4 The Global Fund to fight AIDS, Tuberculosis and Malaria, Geneva, Switzerland; Tulane University School of Public Health and Tropical Medicine, UNITED STATES

## Abstract

**Background:**

Retention in anti-retroviral therapy (ART) presents a challenge in sub-Saharan Africa. In Mozambique, after roll-out to peripheral facilities, the 12-month retention rate was reported mostly from sites with an electronic patient tracking system (EPTS), representing only 65% of patients. We conducted a nationally representative study, compared 12-month retention at EPTS and non-EPTS sites, and its predictors.

**Methods:**

Applying a proportionate to population size sampling strategy, we obtained a nationally representative sample of patients who initiated ART between January 2013 and June 2014. We calculated weighted proportions of the patients’ status at 12 months after ART initiation, and 12-month incidence of lost to follow-up (LTFU) and death. We assessed determinants of LTFU and death by calculating adjusted hazard ratios (AHR) through multivariate cox-proportional hazard models.

**Results:**

Among 19,297 patients sampled, 54.3% were still active, 33.1% LTFU, 2.0% dead, 2.6% transferred-out and 8.0% had unknown status, 12 months after ART initiation. Total attrition rate (LTFU or dead) was 45.5/100PY, higher at facilities without EPTS (51.8/100PY) than with EPTS (37.7/100PY). Clinical stage IV (AHR = 1.7), CD4 count ≤150 (AHR = 1.3) and being pregnant (AHR = 1.6) were significantly associated with LTFU. Clinical stage III or IV (AHR = 2.1 and 3.8), CD4 count ≤150 (AHR = 3.0), not being pregnant (AHR = 3.0), and ART regimens with stavudine (AHR = 4.28) were significantly associated with deaths. Patients enrolled in adherence support groups were 4.6 times less likely to be LTFU, but the number (n = 174) was too small to be significant (p = 0.273).

**Conclusion:**

Retention in ART was substantially lower at non-EPTS sites. EPTS should be expanded to all ART sites to facilitate comprehensive routine monitoring of retention in care. Retention in Mozambique is low and needs to be improved, especially among pregnant women and patients with advanced disease at ART initiation. The effect of ART adherence support groups needs to be further monitored.

## Introduction

The delivery of anti-retroviral therapy (ART) has been gradually rolled out in sub-Saharan Africa over the past decade. UNAIDS estimates that ART coverage in Eastern and Southern Africa increased from 24% in 2010 to 54% in 2015, reaching a total of 10.3 million people [1]. However, retention in care remains challenging. Several studies and reports have documented high attrition rates [2–4]. A 2015 review of 123 surveys studying attrition rates in sub-Saharan Africa estimated the 36-month retention rate to be 65% [5]. Causes of high attrition are multi-faceted. They include patient-related factors, such as socio-economic and educational levels, distance living from the ART facility and mobility; health system-related factors, such as providers’ attitudes, waiting times, drug side effects and defaulter tracking systems; and community-level factors, such as level of social and adherence support [6, 7].

Mozambique, a country with an estimated adult HIV prevalence of 12.3% in 2016 [8], has gradually rolled out ART from hospitals to peripheral and smaller health centres. These include two types of urban health centres and three types of rural health centres. Urban health centres A are larger than urban health centres B, but offer mostly the same primary health care services. The smaller type III rural health centres offer only basic care, type II health centres have a maternity and type I centres an in-patient department. The number of facilities offering ART increased from less than 300 in 2011 to 937 in 2016. The number of people on ART increased to 990,085 by December 2016, corresponding with 54% of those in need [9]. At the time of our survey, the criteria to initiate ART in adults and children 5 years or older were WHO clinical stage III or IV, TB co-infection or a CD4 count≤350 cell/mm^3^. Children under-five years of age received universal treatment. The Ministry of Health adopted in 2013 the Option B+ approach, in which pregnant women are immediately offered HIV treatment for life regardless of their CD4 count or clinical stage. It was gradually being rolled-out during the period assessed. In 2015, to enhance retention in care, the country adopted a strategy of ART adherence support groups, based on a pilot that had shown promising results [10–12]. Retention in care is not included in the routine monitoring system of the Ministry of Health, and the country uses the bi-annual indicator generated by the United States President's Emergency Plan for AIDS Relief (PEPFAR). This calculates the percentage of patients still active in care 12 months after initiating ART, using the data from the PEPFAR-supported ART facilities. In December 2014 and 2015 the retention rate reported from these PEPFAR sites was 67% and 66%, respectively [13]. However, facilities not supported by PEPFAR were generally smaller, more peripheral and without electronic patient tracking system (EPTS), from which the indicator could easily be extracted. EPTS had gradually been introduced by different partners in Mozambique over the past years, but had not yet achieved full coverage. Facilities with EPTS represented only about 65% of HIV patients, and the retention rate at non-EPTS facilities was unknown. At the end of 2014 only 232 of the 795 ART facilities (29%) had an EPTS. With the exception of some larger PEPFAR-supported ART facilities in the central region of the country, non-EPTS facilities are smaller and more remote, and the hypothesis was that attrition is higher. The Ministry of Health and its partners therefore decided to conduct a study with the objective to estimate 12-month retention in care at non-EPTS sites, compare it to the retention rate at EPTS sites, and define the national level ART retention rate. To our knowledge, this is the first study conducting such a comparison. In addition, the study assessed factors associated with attrition, with a particular focus on the effect of recent developments such as the expansion of Option-B plus and the ART adherence support groups. Attrition rates have shown to be influenced by these developments and few studies assessed their effect on a national scale. The findings were compared to those from previous studies in Mozambique to detect any trends.

## Methods

We retrospectively analysed the routinely collected data of a nationally representative sample of HIV patients who initiated ART between January 1, 2013 and June 30, 2014. For this purpose, we selected 58 facilities applying a two-stage probability proportional to population size sampling approach, of which 18 had an EPTS and 40 had no EPTS. At the EPTS facilities, we used the data from the EPTS database. At the non-EPTS facilities, we extracted data from the paper-based reporting tools, namely ART register books, patient charts, and individual patient anti-retroviral (ARV) pharmacy refill cards.

**Fig 1:**
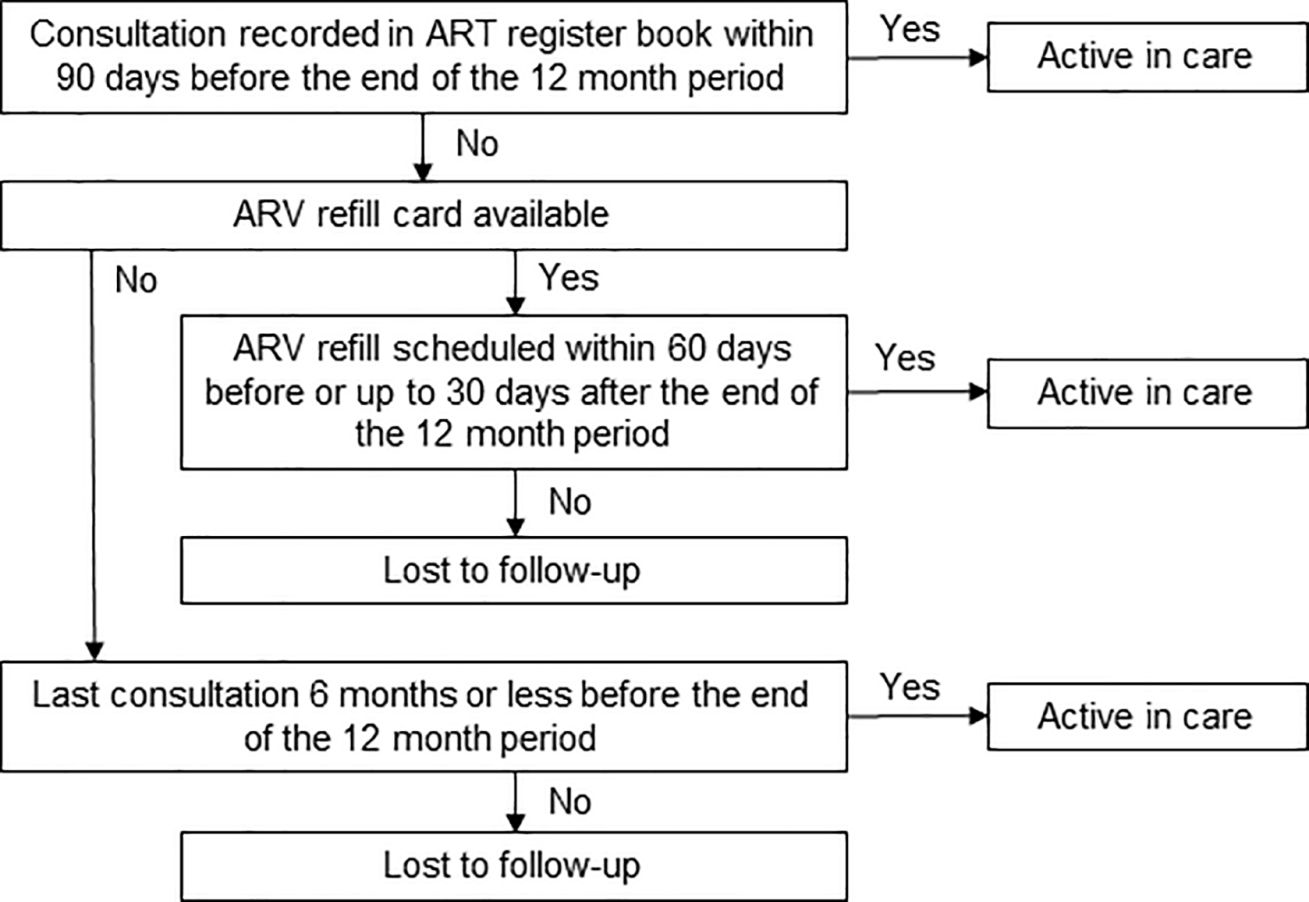
Patient outcome classification process at non-EPTS facilities.

The patient’s status 12 months after having initiated ART was established by analysing the date of initiation, the dates of consultation, the dates of ARV collection, the dates of planned ARV collection and the recorded outcomes. We classified patient outcomes as ‘dead’, ‘transferred-out’ or ‘stopped treatment’, if they had this outcome recorded during the 12-month period, and if the date was consistent with the dates of last consultation and ARV refill. The Ministry of Health defined stopped treatment as ‘stopped due the following factors: drug toxicity, adverse drug reaction, etc.’. We considered patients as ‘lost to follow-up’ (LTFU) if they had not been classified as dead, transferred-out or stopped treatment, and had no ARV refill scheduled within 60 days before or up to 30 days after the end of the 12-month period. At non-EPTS facilities, we considered all patients who had a consultation recorded within 90 days before the end of the 12 months period as active in care, without consulting the ARV refill card. We assumed that the patient collected ARV after consultation and had an ARV refill scheduled 30 days later. Of patients who had no consultation date within 90 days before the end of the period, we consulted the ARV refill card and checked the scheduled refill date. Patients for whom it was not possible to retrieve the ARV refill card, and for whom we could therefore not establish the status with certainty, were classified as ‘status uncertain’. Because at non-EPTS facilities this occurred frequently, and to enable comparison between EPTS and non-EPTS facilities, we performed a second round of classification. We considered these patients active in care if their last consultation was 6 months or less before the end of the period, and LTFU if it was more than 6 months before the end of the period. Patients for whom no last consultation and ARV collection dates were available, and patients with inconsistent dates were classified as ‘status unknown’. The classification process at non-EPTS sites is schematically presented in Fig 1.

We compared the characteristics of the sampled facilities with all ART facilities in the country in terms of province, urban or rural location, type of health facility, and the number of people on ART. We then applied weights to adjust for the over and under-representation of these characteristics. Weighted point estimates for patients who were active in care, had died, were transferred out, had stopped treatment and were lost to follow-up, 12 months after having initiated ART, were calculated with their 95% confidence intervals. We used bootstrapping and adjusted for the cluster effect in patients assessed at the same facility. To estimate incidence rates of lost to follow-up and death, the total amount of exposed person-months was calculated by subtracting the date of initiation of ART from the date of outcome. We used a Poisson regression model, excluding patients who were transferred out or for whom the status was, after the final classification, unknown. To assess if lost to follow-up and death were independently associated with the collected patient or facility characteristics, we applied a multivariate cox-proportional hazard model, using jack-knife resampling and controlling for the cluster effect. We used a forward stepwise approach, adding potential confounding characteristics to the model and withholding them if they altered the hazard ratio (HR) with more than 5%. Patients with a missing value for the characteristic were included as a separate category. The variable EPTS/non-EPTS facility was systematically included in all models. We excluded patients with an uncertain outcome in this analysis. We used STATA IC 14.0 (StataCorp; College Station, TX) for all analyses.

All patient names or other personal identifiers recorded during data collection were removed and the analysis was conducted on a fully anonymized dataset. The final database was password protected. The study protocol was approved by the National Committee of Bioethics for Health in Mozambique.

## Results

Table 1 presents the characteristics of the 19,297 patients enrolled in ART between January 2013-June 2014 at the 58 sampled facilities, of which 11,227 (58%) had enrolled at facilities with an EPTS and 8,070 (42%) at a facility without EPTS. Most patients were enrolled at rural health centres, in particular of type II (38%) and type I (23%), followed by urban health centres B (18%) and hospitals (15%). The sample did not contain any urban health centre A. Most facilities without EPTS (29/40) were smaller sites with less than 400 patients enrolled, and patients at facilities with EPTS were therefore more often from large ART sites (38% at facilities with more than 2000 patients on ART at the end of 2014) than patients at non-EPTS sites (6%). Most patients were adults (83%) and female (72%). The most prevalent age category was between 26 and 35 years old (34%). Of the adults for whom data were available for marital status, 54% were either married or cohabiting. WHO clinical stage data at ART initiation were available for 87% of patients and CD4 count data for only 58%. Of patients with a recorded clinical stage, 60% percent were in stage I, and of patients with a CD4 result, 33% had a count of 350 or more. Approximately one third (32%) of the adult women were pregnant at ART initiation, and of these 81% were recorded as having initiated under option B+. Six percent were recorded as being co-infected with tuberculosis. The most common ART regimen initiated was the first-line treatment for adults and children≥5 years, 3TC+TDF+EFV (47%). About one third (31%) was initiated with the other adult first-line treatment, and first-line treatment in children under-five, 3TC+AZT+NVP, and 11% with 3TC+AZT+EFV, the alternative given in case of renal insufficiency or diabetes. Only 174 patients (0.9%) had participated in an ART adherence support group.

**Table 1:** Patient characteristics by EPTS/non-EPTS facility (N = 19,297).

	EPTS facilities (N = 11227)		Non-EPTS facilities (N = 8070)		Total (N = 19297)	
Characteristic	n	%	n	%	n	%
*Type of facility*						
Rural Health Centre type I	2313	20.6	2150	26.6	4463	23.1
Rural Health Centre type II	2722	24.3	4655	57.7	7377	38.2
Rural Health Centre type III	1074	9.6	0	0.0	1074	5.6
Urban Health Centre type B	3095	27.6	408	5.1	3503	18.2
District Hospital	0	0.0	501	6.2	501	2.6
Rural Hospital	0	0.0	356	4.4	356	1.8
Provincial Hospital	359	3.2	0	0.0	359	1.9
General Hospital	1477	13.2	0	0.0	1477	7.7
Central Hospital	187	1.7	0	0.0	187	1.0
*Size of facility (No on ART at the end of 2014)*						
Less than 400	1602	14.3	2963	36.7	4565	23.7
400–999	2524	22.5	2110	26.2	4634	24.0
1000–1999	2820	25.1	2496	30.9	5316	27.6
2000 or more	4281	38.1	501	6.2	4782	24.8
*Age*						
< = 5	655	5.8	441	5.5	1096	5.7
6–15	340	3.0	139	1.7	479	2.5
16–25	2224	19.8	2072	25.7	4296	22.3
26–35	4279	38.1	2269	28.1	6548	33.9
36–45	2179	19.4	887	11.0	3066	15.9
>45	1548	13.8	476	5.9	2024	10.5
No information	2	0.0	1786	22.1	1788	9.3
*Adult/child*						
Adult (> = 15 years)	10266	91.4	5737	71.1	16003	82.9
Child (<15 years)	959	8.5	549	6.8	1508	7.8
No information	2	0.0	1784	22.1	1786	9.3
*Sex*						
Female	7931	70.6	5979	74.1	1391	72.1
Male	3296	29.4	2091	25.9	5387	27.9
*Marital status*^*1*^	*(N = 10266)*		*(N = 5737)*		*(N = 16003)*	
Married	1195	11.6	1138	19.8	2333	14.6
Living with partner	3173	30.9	1444	25.2	4617	28.9
Single	3854	37.5	1230	21.4	5084	31.8
Widowed/Separated	537	5.2	216	3.8	753	4.7
No information	1507	14.7	1709	29.8	3216	20.1
*WHO clinical stage*						
Stage I	4851	43.2	3172	39.3	8023	41.6
Stage II	1954	17.4	1446	17.9	34	17.6
Stage III	2201	19.6	2221	27.5	4422	22.9
Stage IV	566	5.0	415	5.1	981	5.1
No information	1655	14.7	816	10.1	2471	12.8
*CD4 count*						
<50	601	5.4	292	3.6	893	4.6
50–199	1766	15.7	1210	15.0	2976	15.4
200–349	2084	18.6	1537	19.1	3621	18.8
350–499	867	7.7	760	9.4	1627	8.4
> = 500	1033	9.2	1095	13.6	2128	11.0
No information	4876	43.4	3176	39.4	8052	41.7
*Pregnant*^*2*^	*(N = 7425)*		*(N = 4333)*		*(N = 11758)*	
No	5861	78.9	2090	48.2	7951	67.6
Yes	1553	20.9	2170	50.1	3723	31.7
No information	11	0.2	73	1.7	84	0.7
*Option B+*^*3*^	*(N = 1556)*		*(N = 2677)*		*(N = 4233)*	
No	273	17.5	26	1.0	299	7.1
Yes	811	52.1	2630	98.2	3441	81.3
No information	472	30.3	21	0.8	493	11.7
*TB infection*						
No	9707	86.5	7220	89.5	16927	87.7
Yes	660	5.9	415	5.1	1075	5.6
No information	860	7.7	435	5.4	1295	6.7
*ART regimen at ART initiation*						
3TC+TDF+EFV	6463	57.6	2683	33.3	9146	47.4
3TC+AZT+NVP	2960	26.4	3078	38.1	6038	31.3
3TC+AZT+EFV	564	5.0	1498	18.6	2062	10.7
3TC+D4T+NVP	704	6.3	444	5.5	1148	6.0
other	232	2.1	132	1.6	364	1.9
No information	304	2.7	235	2.9	539	2.8
*Participated in ART adherence support group*						
No	11155	99.4	7625	94.5	18780	97.3
Yes	72	0.6	102	1.3	174	0.9
No information	0	0.0	343	4.3	343	1.8

^1^N = adult

^2^N = Female patients> = 15 years

^3^N = Pregnant women

We could establish with certainty the status 12 months after ART initiation for almost all patients from EPTS facilities (99.0%), but for only 65% of patients at non-EPTS facilities. The 2,935 patients with an uncertain status were relatively more often from rural health centres type II (66.1%) than those with certain status (33.2%), but had otherwise similar characteristics. Based on the criteria described above, we classified 1,541 of the patients with an uncertain status as lost to follow-up, and 261 patients as active in care, leaving 1,133 patients (6%) without sufficient information classified as ‘unknown status’.

Table 2 presents the proportions of the patients’ status 12 months after ART initiation, weighted for facility characteristics. In the final classification, slightly more than half of the patients (54%) were confirmed to have been still active in care, 2.6% were transferred out, 2.0% had died, 33% were lost to follow-up (LTFU) and for 8% the status was unknown. No patient was recorded as having stopped treatment. Excluding patients whose status was unknown or were transferred out, this translated into a death rate of 2.6/100PY and a LTFU rate of 42.9/100PY, and thus a total attrition rate ((LTFU or dead) of 45.5/100PY (Table 3). Attrition was substantially higher at non-EPTS sites (51.8/100PY), than at EPTS sites (37.7/100PY).

**Table 2:** Patients’ status 12 months after ART initiation by type of facility (N = 19,927).

Status	EPTS facilities (N = 11227)		Non-EPTS facilities (N = 8070)		Total (N = 19297)	
	Weighted %	95% CI	Weighted %	95% CI	Weighted %	95% CI
*Initial classification*						
Active	64.7	56.4–73.0	44.0	39.7–48.3	52.8	41.8–63.7
Lost to follow-up	27.0	22.7–31.3	22.1	17.3–27.0	24.2	21.8–26.6
Death	3.5	1.3–5.7	0.9	0.0–2.6	2.0	0.4–3.6
Transferred out	4.0	0.9–7.1	1.6	0.2–2.9	2.6	0.9–4.2
Uncertain outcome	1.0	0.0–2.0	31.4	27.3–35.5	18.5	5.2–32.8
*Final classification*						
Active	64.7	56.4–73.0	46.7	42.2–51.2	54.3	44.2–64.4
Lost to follow-up	27.2	22.9–31.4	37.5	30.6–44.4	33.1	26.1–40.1
Death	3.5	1.3–5.7	0.9	0.0–2.6	2.0	0.4–3.6
Transferred out	4.0	0.9–7.1	1.6	0.2–2.9	2.6	0.9–4.2
Unknown outcome	0.7	0.0–1.5	13.4	11.2–15.6	8.0	2.6–13.4

**Table 3:** Attrition rate by type of facility (PY = 17,068).

Outcome	EPTS facilities (Person-Years = 7539)					Non-EPTS facilities (Person-Years = 9529)			Total (Person-Years = 17068)		
	n		n/100 PY		95% CI	n	n/100 PY	95% CI	n	n/100 PY	95% CI
Lost to follow-up	2568	33.4		28.1–39.7		4841	50.5	44.8–57.0	7409	42.9	35.1–52.5
Death	329	4.3		2.8–6.5		115	1.2	0.3–3.9	444	2.6	1.4–4.6
Total attrition	2897	37.7		31.8–44.8		4956	51.8	46.6–57.4	7853	45.5	38.5–53.8

Table 4 and Table 5 present the results of the multivariate analysis, applying a cox-proportional hazard model, assessing determinants of LTFU and death. After adjusting for the confounding effect of other available determinants, being pregnant was still significantly (p<0.05) associated with LTFU. Patients at smaller ART sites were 1.61 times more likely to be LTFU (p = 0.088). Clinical stage, pregnancy, ART regimen, sex and CD4 count were all associated with death. Stage IV patients were 3.8 times more likely and Stage III patients 2.1 times more likely to have died than patients in Stage I. Non-pregnant patients were 3.2 times more at risk than pregnant patients. Patients enrolled with 3TC+D4T+NVP had a 3.1 times higher probability and patients with regimens other than 3TC+TDF+EFV, 3TC+AZT+EFV, 3TC+AZT+NVP or 3TC+D4T+NVP a 3.2 times higher probability to have died than patients enrolled with 3TC+TDF+EFV. Male patients were 1.8 times more likely to die than female patients. Patients with CD4≤150 were 3.0 times more at risk of dying than patients with CD4>300.

**Table 4:** Determinants of lost to follow-up (N = 15,750)*.

Characteristic	Crude HR	Adjusted HR	95% CI	p-value
Clinical stage at ART initiation				
Stage I/II/III	-	-	-	-
Stage IV	1.45	1.72	1.35–2.19	<0.001
CD4 count at ART initiation				
151–400	-	-	-	-
150 or less	1.27	1.28	1.07–1.52	0.007
More than 400	1.35	1.06	0.92–1.22	0.435
Pregnant at ART initiation				
No	-	-	-	-
Yes	1.82	1.63	1.34–1.97	<0.001
Option B+				
No	-	-	-	-
Yes	1.94	0.95	0.66–1.36	0.779
Participated in ART support group				
Yes	-	-	-	-
No	8.81	7.09	1.28–39.16	0.025
Participated in ART support group**				
Yes	-	-	-	-
No	5.18	4.56	0.29–70.64	0.273
No on ART at the end of 2014				
More than 1000	-	-	-	-
Less than 1000	1.61	1.47	0.94–2.31	0.088

* Patients transferred out or of whom the outcome could not be ascertained with certainty were excluded. Patients who died are included.

** Excluding patients who were no longer active at 6 months.

**Table 5:** Determinants of deaths (N = 15,750)*.

Characteristic	Crude HR	Adjusted HR	95% CI	p-value
Sex				
Female	-	-	-	-
Male	2.86	1.83	1.50–2.22	<0.001
Clinical stage at ART initiation				
Stage I	-	-	-	-
Stage II	1.72	1.12	0.81–1.57	0.485
Stage III	3.51	2.10	1.44–3.05	<0.001
Stage IV	7.18	3.82	2.50–5.83	<0.001
CD4 count at ART initiation				
>300	-	-	-	-
151–300	1.48	1.26	0.81–1.96	0.295
< = 150	3.59	3.01	1.84–4.91	<0.001
Pregnant at ART initiation				
Yes	-	-	-	-
No	7.98	3.19	1.58–6.45	0.002
Option B+				
Yes	-	-	-	-
No	13.4	1.95	0.29–12.95	0.481
ART Regimen at ART initiation				
3TC+TDF+EFV	-	-	-	-
3TC+AZT+EFV	1.89	1.89	1.09–3.27	0.024
3TC+AZT+NVP	2.20	2.31	1.19–4.48	0.014
3TC+D4T+NVP	4.28	3.13	1.57–6.23	0.002
Other	4.88	3.24	1.74–6.02	<0.001

* Patients transferred out or of whom the outcome could not be ascertained with certainty were excluded

## Discussion

This study is the first nationally representative study on ART retention and its predictors in Mozambique, since the implementation of the national acceleration plan and the scaling up of ART to more peripheral facilities [14]. The analysis clearly demonstrated that retention in antiretroviral treatment was low overall. The 54% retention on ART at 12 months in our study is lower than the global average of 74% and the WHO-recommended target of 85% [15]. The retention rate was lower than what was routinely reported by PEPFAR-supported facilities. The facilities that do not report retention through PEPFAR are all non-EPTS facilities, which had lower retention on treatment. They have on average a smaller number of people on ART, which was also correlated with LTFU. Our study indicates a need to include all ART facilities in the routine monitoring of national retention rates, and not only those where data can easily be accessed. This is best achieved by introducing an EPTS at as many ART facilities as possible [16].

Our national attrition rate estimate of 45.5/100 PY was slightly higher compared to the results of a retrospective cohort study done in Central Mozambique in 2006–2008 [17] and substantially higher than what was found in two other studies in 2002–2006 and 2005–2011, respectively [18, 19]. These studies were however not nationally representative. A similar retrospective analysis of a nationally representative sample of 2,596 adults initiating ART at 58 facilities in Mozambique was done during 2004–2007, before the expansion to more peripheral facilities. It found that after one year 5% had died, 15% was lost to follow-up and 1% stopped ART [20]. We observed a much higher LTFU rate and a lower death rate, which might indicate that these changed over the last 10 years. An evaluation of outcome trends among adult ART patients in Mozambique in the period 2004–2013 at 170 facilities with an EPTS, observed that the 1-year LTFU rate increased during that period from 26% to 31% and the mortality declined from 9% to 3%, supporting this conclusion [21]. Over that period, the patient population began to include more female patients, a larger share of whom were pregnant, and to have a higher baseline CD4 count. These three characteristics were negatively associated with death in our analysis, providing a possible explanation for the lower mortality rate. An evidence-based explanation for the increased LTFU rate is harder to find. The increase in the proportion of pregnant women, who have a higher LTFU rate, is probably a contributing factor. It could also be that as the number of people on ART at a facility increases, the more difficult it becomes to track defaulters. Another factor might be the further roll-out of ART to more peripheral facilities where retention in care is more challenging. The number of people on ART is foreseen to further increase with the implementation of the test-and-treat guidelines [22], and extra attention on retention in care is indicated.

The attrition rate that we observed was higher than those found in most other sub-Saharan African countries. Two systematic literature reviews of studies describing retention rates from observational cohorts of adult ART patients in sub-Saharan Africa [3] and one review of one-year retention amongst African children in ART [23], found substantially lower average attrition rates than what we observed. This is consistent with an analysis of attrition rates that was done in four countries (Kenya, Mozambique, Rwanda, and Tanzania) and concluded that Mozambique had the highest attrition, for both adults and children [19, 24].

The clinical status of the patient at ART initiation, measured by the WHO clinical stage and the CD4 count, has been associated with LTFU in other studies. Less healthy patients generally have a higher risk of LTFU [18, 20, 21, 25–27]. This is likely to be partly due to undocumented death among these patients [28]. Some studies found that LTFU also increased among patients with a higher CD4 count [21, 29], but in our study the higher LTFU rate among patients with CD4> = 400 disappeared after adjustment for other factors.

Of particular concern is LTFU among pregnant women that has shown to be a risk factor in other studies [29, 30]. A previous study in Mozambique established that pregnant women had, in the pre-ART period, a higher risk for LTFU [27] and also in the analysis of the treatment outcome of adult ART patients in Mozambique in the period 2004–2013, pregnancy was a risk factor [21]. Particularly, pregnant women enrolled under Option B+, and thus more likely to be healthy, had in this analysis a higher attrition. In our analysis, we found that pregnant women had a much higher LTFU rate than non-pregnant patients, even after controlling for age, sex, clinical stage and other factors. However, there was no significant difference in LTFU rate between pregnant women recorded as initiating ART under Option B+ and other pregnant women, making it uncertain to what extent the Option B+ program plays a role. We observed that the classification of women as initiating treatment under Option B+ differed across facilities, indicating possible misclassification, which could be the explanation for the lack of difference. Retention in care among pregnant women needs to be strengthened, and the effect of the Option B+ program on retention in care further investigated.

A particular interest of the study was to assess the effect of the ART adherence support groups. Having participated in such a group was strongly negatively associated with LTFU. The concept had, however, only recently been introduced and few (174) patients participated in a support group. This substantially reduced the statistical power of our analysis. Patients were only eligible to enrol in such a group when they had been active in care for at least 6 months. We therefore compared patients participating in support groups with other patients who were still active at 6 months, and observed that the LTFU rate was still much higher among non-support group patients (HR 4.56) but, because of the low numbers, the statistical power was too low to rule out the possibility that this was merely due to chance. Also in the study analysing the 2004–2013 EPTS data in Mozambique, participation in a support group was associated with lower LTFU [21]. It is, however, possible that patients who accept to enrol in the support groups are those that are already motivated to adhere to ART. As the program continues to be rolled out, retention in care among patients participating in ART adherence support groups needs to be closely monitored.

Risk factors for dying while in ART have not frequently been studied. The only socio-demographic that remained strongly associated with death, after adjusting for confounders, was gender, with men having an almost double mortality rate than women. This is consistent with what was found in some other studies [25, 31]. An obvious risk factor is the clinical status of the patient at ART initiation, as was documented by several other studies [21, 25, 29, 31, 32]. Both the WHO stage and the CD4 count were strongly and independently associated with death. The higher death rate among patients with more advanced disease indicates a need to improve early detection and enrolment in ART of patients with HIV.

We found a strong negative association between pregnancy at ART initiation and death, similar to what has been documented by some other studies [21, 29]. This could be because most pregnant women were enrolled under Option B+ and were therefore healthier, but even after controlling for clinical stage and CD4 count, the association remained very strong. A possible explanation is that the available clinical information was insufficient to adequately control for all clinical factors and that these factors caused residual confounding.

Finally, the ART regimen was strongly and independently associated with death. In particular regimens containing stavudine (D4T) had a higher mortality rate. The risk of stavudine has previously been demonstrated in several other studies [21, 33, 34] and WHO therefore recommends to phase it out [35]. Also in Mozambique stavudine has been replaced by AZT, but is still used in certain therapeutic regimens such as when AZT causes severe anaemia or in young children co-infected with TB. The presence of stavudine in these regimens could be an explanation for the higher mortality, although that the worse clinical condition of patients using these regimens also could. We did not collect all clinical data, such as for example the haemoglobin status, and could therefore not control for it in our analysis.

The study design was an analysis of data retrospectively collected from routine health facility records and therefore suffers from the limitations inherent to this design. The recording of routine data might not always be precise, which was confirmed by the inconsistencies found, such as dates that do not follow a logical order, contradictory data in different fields, etc. Information was often not filled out and many of the measured variables had a high proportion of missing values. A particular challenge in our study was that it was not always possible to locate and review all source documents. More specifically, at non-EPTS sites we could not access the ARV medicines refill card for 35% of patients. We could therefore not confirm with certainty if patients who had not come for a consultation were lost to follow-up, or if these patients had come to collect ARVs, without coming to consultation. Most of these patients (86%) were patients whose last consultation date was more than six months before the end of the 12-month period, and for 68% more than nine months. The probability that these were patients that had been LTFU was high and we therefore classified them as such. The remaining 14% had come for consultation in the six months preceding the end of the 12-month period, and were presumed to have been patients still active in care. This has without doubt introduced a certain level of misclassification, but we believe not to the extent that it invalidates our findings and conclusions. The bias is most probably towards an underestimation of the attrition rate at non-EPTS facilities. We considered all patients that had come for a consultation between 3 and 6 months before the end of the period as active in care, which might be too optimistic. The difference in retention rate between EPTS and non-EPTS sites might thus be still higher than what we estimated. The fact that it was so difficult to establish with certainty the outcome status for so many patients on ART at facilities without EPTS is of concern. Improving patient follow-up and outcome monitoring practices, for example by introducing EPTS, should be an immediate priority. We collected data applying two different methods, extracting it from EPTS databases and from paper-based recording tools, which reduces to some extent the comparison of EPTS sites with non-EPTS sites. We can, for example, not rule out the possibility that the large difference found in characteristics such as being pregnant and being enrolled in Option B+ is due to a different classification in the two systems.

An important limitation in assessing determinants of attrition is the fact that we only had access to those characteristics available in the routine recording instruments. We don’t know about other factors that have shown to be related to attrition in the sub-Saharan African context, such as patients’ socio-economic and educational level, distance living from ART facility, mobility, providers’ attitudes, level of social support or home based care [4, 5, 22, 23, 24]. Some of these factors might be associated with our measured predictors, and thus be confounders. We can therefore not exclude that some of the established associations are in fact caused by some of these other underlying factors.

## Conclusion

Retention in antiretroviral therapy was very low in Mozambique in the study period, especially among pregnant women and patients with a poor clinical condition at ART initiation, and it should be improved. Facilities without EPTS, not included in the routine monitoring of retention, had a much lower retention rate and routine reporting should include these facilities. Early detection and enrolment in care of people living with HIV needs to be increased to reduce the number of deaths. Patients participating in ART adherence support groups present a promising way forward, despite insignificance in this study due to a small sample. Adherence support needs to be closely monitored to assess what the effect is as the support groups are being rolled out.

## Supporting information

S1 Appendix Cox proportional hazard models. (DOCX)

S1 Dataset (XLS)
