# Supplementary material for: Validity of reported retention in antiretroviral therapy after roll-out to peripheral facilities in Mozambique: Results of a retrospective national cohort analysis
Source: PLoS One. 2018 Jun 21;13(6):e0198916. doi: 10.1371/journal.pone.0198916 (PMC6013210; doi:10.1371/journal.pone.0198916)
Supplement: S1 Appendix — (DOCX) [file pone.0198916.s001.docx]

*Final models used

*Lost to follow-up

stset period2 if final2!=5, failure(lost2==1) scale(1)

stcox age_bi EPTS i.CD400b pregn2 bplus2 i.prov2 i.part if final2!=5, vce(jackknife, cluster(designacao))

stcox sex2 EPTS age_bi pregn2 bplus2 i.CD400b if final2!=5, vce(jackknife, cluster(designacao))

stcox widowed EPTS age_bi pregn2 i.CD400b if final2!=5, vce(jackknife, cluster(designacao))

stcox stage4 EPTS pregn2 i.HF i.bplus2b i.CD400b age_bi if final2!=5, vce(jackknife, cluster(designacao))

stcox i.CD4_6 EPTS pregn2 bplus2 i.ARTREG i.prov2 i.HF i.part size2 if final2!=5, vce(jackknife, cluster(designacao))

stcox pregn2 EPTS age_bi sex i.CD400b i.HF i.part if final2!=5, vce(jackknife, cluster(designacao))

stcox bplus2 EPTS pregn2 i.CD400b i.prov2 i.HF i.part size2 if final2!=5, vce(jackknife, cluster(designacao))

stcox TB EPTS age_bi i.CD400b pregn2 i.ARTREG3 if final2!=5, vce(jackknife, cluster(designacao))

stcox i.ARTREG2 EPTS age_bi i.CD400b pregn2 TB i.HF size2 if final2!=5, vce(jackknife, cluster(designacao))

stcox nogaac EPTS age_bi i.CD400b pregn2 i.prov2 i.part if final2!=5, vce(jackknife, cluster(designacao))

stcox nogaac EPTS age_bi i.CD400b pregn2 i.prov2 i.part if final2!=5 & period>182.5, vce(jackknife, cluster(designacao))

stcox i.HF EPTS i.CD400b pregn2 TB i.prov2 i.part size2 if final2!=5, vce(jackknife, cluster(designacao))

stcox size2 EPTS age_bi i.CD400b i.prov2 i.part i.HF if final2!=5, vce(jackknife, cluster(designacao))

*deceased

stset period2 if final2!=5, failure(deceased==1) scale(1)

stcox i.age4 sex2 i.WHOstage i.CD4_3 TB i.ARTREG i.prov4 HF2 part2 NOMCH EPTS if final2!=5, vce(jackknife, cluster(designacao))

stcox i.age4 sex2 i.WHOstageb i.CD4_3b pregn3 i.ARTREG i.prov4 HF2 EPTS if final2!=5, vce(jackknife, cluster(designacao))

stcox child sex2 i.WHOstageb i.CD4_3b pregn3 i.ARTREG i.prov4 i.HF3 EPTS if final2!=5, vce(jackknife, cluster(designacao))

stcox sex2 EPTS i.NOBP if final2!=5, vce(jackknife, cluster(designacao))

stcox sex2 EPTS i.NOMCH if final2!=5, vce(jackknife, cluster(designacao))

stcox sex2 i.age4b i.WHOstageb i.CD4_3b pregn3 i.prov4 EPTS if final2!=5, vce(jackknife, cluster(designacao))

stcox i.marital3 EPTS i.age4b sex2 pregn3 TBb i.ARTREG HF2 if final2!=5, vce(jackknife, cluster(designacao))

stcox i.marital3 EPTS i.age4b sex2 pregn3 TBb i.ARTREG HF2 i.WHOstageb i.CD4_3b if final2!=5, vce(jackknife, cluster(designacao))

stcox i.WHOstage EPTS i.age4b sex2 i.marital3b i.CD4_3b pregn3 i.ARTREG i.prov4 HF2 i.NOBP if final2!=5, vce(jackknife, cluster(designacao))

stcox i.CD4_3 EPTS i.age4b sex2 pregn3 i.ARTREG part2 HF2 i.NOBP i.NOMCH if final2!=5, vce(jackknife, cluster(designacao))

stcox pregn3 EPTS i.age4b sex2 i.marital3b i.WHOstageb i.ARTREG i.prov4 HF2 if final2!=5, vce(jackknife, cluster(designacao))

stcox pregn3 EPTS i.age4b sex2 i.marital3b i.WHOstageb i.ARTREG i.prov4 HF2 TBb i.CD4_3b if final2!=5, vce(jackknife, cluster(designacao))

stcox bplus3 if final2!=5, vce(jackknife, cluster(designacao))

stcox bplus3 i.age4b sex2 i.marital3b i.WHOstageb pregn3 i.ARTREG i.prov4 HF2 i.NOBP EPTS if final2!=5, vce(jackknife, cluster(designacao))

stcox TB sex2 i.WHOstageb i.CD4_3b pregn3 i.ARTREG HF2 i.NOMCH EPTS if final2!=5, vce(jackknife, cluster(designacao))

stcox i.ARTREG i.age4b sex2 i.WHOstageb pregn3 TBb i.prov4 HF2 i.NOBP i.NOMCH EPTS if final2!=5, vce(jackknife, cluster(designacao))

stcox i.ARTREG i.CD4_3b i.age4b sex2 i.WHOstageb pregn3 TBb i.prov4 HF2 i.NOBP EPTS i.marital3b if final2!=5, vce(jackknife, cluster(designacao))

stcox i.prov4 i.CD4_3b i.age4b sex2 i.WHOstageb pregn3 i.ARTREG TBb i.prov4 HF2 i.NOMCH EPTS i.marital3b if final2!=5, vce(jackknife, cluster(designacao))

stcox HF2 i.CD4_3b i.age4b i.WHOstageb i.prov4 i.marital3b EPTS if final2!=5, vce(jackknife, cluster(designacao))

stcox part2 i.CD4_3b i.ARTREG TBb i.NOMCH EPTS i.marital3b if final2!=5, vce(jackknife, cluster(designacao))

stcox NOBP sex2 i.WHOstageb pregn3 TBb i.prov4 HF2 EPTS if final2!=5, vce(jackknife, cluster(designacao))

stcox NOMCH i.age4 sex2 i.CD4_3b i.WHOstageb pregn3 TBb i.prov4 HF2 EPTS if final2!=5, vce(jackknife, cluster(designacao))
